# Capsaicin Functions as *Drosophila* Ovipositional Repellent and Causes Intestinal Dysplasia

**DOI:** 10.1038/s41598-020-66900-2

**Published:** 2020-06-19

**Authors:** Yaoxing Li, Peng Bai, Longsheng Wei, Ruxue Kang, Lirong Chen, Mingliang Zhang, Eng King Tan, Wei Liu

**Affiliations:** 1 Department of Clinical Medical, Fenyang College, Shanxi Medical University, Shanxi, China; 2 Department of Basic Medical, Fenyang College, Shanxi Medical University, Shanxi, China; 3 Department of Medical Laboratory Science, Fenyang College, Shanxi Medical University, Shanxi, China; 4 Department of Neurology, National Neuroscience Institute, Singapore General Hospital Campus, Singapore, Singapore

**Keywords:** Social behaviour, Stress and resilience

## Abstract

Plants generate a plethora of secondary compounds (toxins) that potently influence the breadth of the breeding niches of animals, including *Drosophila*. Capsaicin is an alkaloid irritant from hot chili peppers, and can act as a deterrent to affect animal behaviors, such as egg laying choice. However, the mechanism underlying this ovipositional avoidance remains unknown. Here, we report that *Drosophila* females exhibit a robust ovipositional aversion to capsaicin. First, we found that females were robustly repelled from laying eggs on capsaicin-containing sites. Second, genetic manipulations show that the ovipositional aversion to capsaicin is mediated by activation of nociceptive neurons expressing the *painless* gene. Finally, we found that capsaicin compromised the health and lifespan of flies through intestinal dysplasia and oxidative innate immunity. Overall, our study suggests that egg-laying sensation converts capsaicin into an aversive behavior for female *Drosophila*, mirroring an adaptation to facilitate the survival and fitness of both parents and offspring.

## Introduction

The environment contains a variety of threats to inhabitants, and many plants generate toxins that influence the breadth of the breeding niche of animals^1^. Capsaicin (8-methyl-N-vanillyl-6-nonenamide) is a major pungent component in chili peppers. It exerts potent effects on numerous physiological processes in animals^2^. Accumulating evidence suggests that capsaicin causes a sensation of burning pain through chemoreceptors and nociceptors^3,4^. Accordingly, it acts as an irritant for many species ranging from insects to mammals. Extensive studies have shown that capsaicin affects foraging, food-averse migratory behavior, and social behavior in insects^5–7^. For instance, capsaicin inhibits the foraging of the beetle (*Tenebrio molitor*)^8^. Any inhabitants must use elaborate defensive mechanisms to protect against capsaicin, because overcoming this antagonism is critical for survival. Notably, several studies have reported that capsaicin acts as a repellent that affects the egg-laying decisions of several insects^8,9^. However, this behavior and the mechanism underlying how capsaicin causes contact- or ingestion-dependent pathogenesis in flies remain to be thoroughly investigated in an ecological context. The vinegar fly, *Drosophila melanogaster*, mainly breeds on decaying fruits and continuously explores favorite substrates prior to depositing each egg. The egg-laying behavior is an innate behavior for *Drosophila* propagation^10,11^, making *D. melanogaster* as a feasible model for investigating the effect of capsaicin on this behavior. Female egg-laying involves a complex assessment of cues regarding the opportunities and threats in the surroundings^11^. Given that detecting danger is a primary task for their survival and reproduction, *Drosophila* females are sensitive to potentially toxic substrates, such as pathogens^12,13^, wasps^14^, and alkaline substances^15^. Nevertheless, whether *Drosophila* is repelled from oviposition by the capsaicin remains unknown.

The adult fly gut, similar to the mammalian gut, is a plastic and functionally compartmentalized organ lined by epithelia^16,17^. The intestinal epithelia form a gut barrier that allows the gut to digest and absorb nutrients but restricts host contact with various xenobiotics. Concurrently, the gut tolerates a variety of stresses and is susceptible to acute and chronic toxicants. Stress results in intestinal epithelium impairment and intestinal barrier dysfunction in mammals and *Drosophila*. Thus, capsaicin has been postulated to accelerate the onset of intestinal barrier defects and shorten the lifespan^18^. To maintain intestine integrity, intestinal epithelia undergo stress-induced turnover throughout the lifespan. In the adult midgut, intestinal stem cells are the sole dividing cells, and they generate two differentiated intestinal cell types: enteroendocrine cells and enterocytes^18^. This regeneration makes the *Drosophila* intestine a promising model for deciphering stress-related alterations in innate immune signaling and regenerative capacity. Studies have found that dual oxidase (*Duox*)-mediated production of reactive oxygen species (ROS) is a primary immune mechanism underlying intestinal homeostasis^19,20^. However, the mechanism through which immune activation leads to capsaicin-related pathologies and lifespan impairment is not fully understood.

In the current study, we expected that capsaicin would induce an ovipositional avoidance of *Drosophila melanogaster*. To pursue this hypothesis, we used this model organism to explore the roles of capsaicin in oviposition decisions and examined how capsaicin affects the lifespan of *Drosophila*, providing insight into an adaptation to facilitate the survival and propagation of flies.

## Results

### Egg-laying avoidance to capsaicin

**Figure 1:**
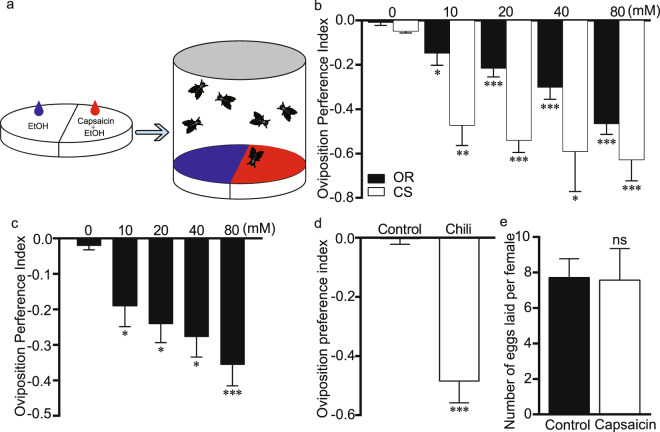
*Drosophila* ovipositional aversion to capsaicin. (**a**) Schematic of the two-choice assay used to assess egg-laying preferences of *Drosophila*. Female flies were allowed to lay eggs in a dish in which one half of the food contained capsaicin, and the other contained ethanol. The numbers of eggs were counted on each half, and the oviposition preference index was calculated. (**b**) The ovipositional aversion to capsaicin substrate by wild-type Oregon R (OR) and Canton S (CS) flies. (**c**) The ovipositional aversion to capsaicin substrate by *D. yakuba*. (**d**) The quantification of ovipositional aversion to chili juice. (**e**) Capsaicin did not hinder the number of egg-laying in the whole-forced substrate. The average number of eggs laid by female was calculated. The one-sample *t*-test was used to assess the mean deviation of each column from 0 mM. n = 8–15, 3 replicates. Mean ± S.E.M.; symbols: NS p > 0.05; *p < 0.05; **p < 0.01; ***p < 0.001.

To investigate how gravid females respond to capsaicin, we evaluated their egg-laying preferences by using a two-choice apparatus^10,21^ (Fig. 1a). Interestingly, we found that Oregon R (OR) females used as wild-type flies significantly displayed ovipositional avoidance of capsaicin in a dose-dependent manner (Fig. 1b). For instance, OR females laid approximately 35% of their eggs on food halves with capsaicin, and the oviposition index (OI) was −0.46 (Fig. 1b). This result suggested that females were prone to avoiding laying eggs in response to capsaicin. In addition, another wild-type Canton S fly similarly showed oviposition repellence to 80 mM capsaicin, with an OI of −0.63 (Fig. 1b), thus suggesting that genetic variation did not account for this oviposition preference. Since two strains of Drosophila similarly responded to capsaicin, a representative Oregon R was used in our later experiments. Moreover, another closely related *Drosophila* species, *D. yakuba*, exhibited an aversion to laying eggs on capsaicin-containing substances (Fig. 1c), implicating that *Drosophila* has evolved a conserved capability of discriminate capsaicin prior to selecting oviposition sites. Interestingly, we also observed that Thailand chili repelled oviposition in OR females (Fig. 1d). Notably, capsaicin did not inhibit egg laying in general, because OR flies laid comparable numbers of eggs in the absence or presence of 40 mM capsaicin (Fig. 1e). This result suggests that the robustly negative oviposition preference for capsaicin is not simply attributable to a capsaicin-induced decrease in egg laying but instead represents an active choice made by female flies to avoid a repulsive substrate^21^. Together, our results demonstrated that capsaicin triggered ovipositional avoidance in *Drosophila*.

### Positional and feeding avoidance of capsaicin

**Figure 2:**
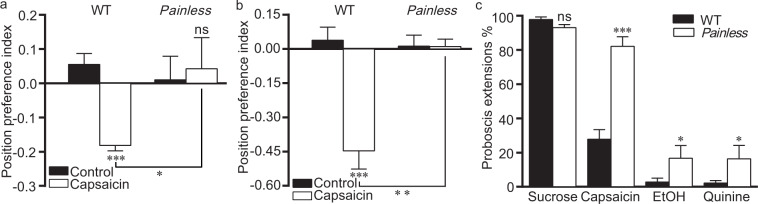
Positional and feeding avoidance of capsaicin by *Drosophila*. (**a,b**) The positional aversion of adults to capsaicin. (**a**) Wild-type adult female flies were averse to the capsaicin substrate, whereas *Painless* mutants showed neutral responses. The one-sample *t*-test was used to assess the mean deviation of each column from 0; 20 mM capsaicin was used. n = 150. (**b**) The positional aversion of wild-type larvae to capsaicin. Larvae were presented with a two-choice agar plate; 20 mM capsaicin was used, n = 10. (**c**) The feeding aversion to capsaicin. Wild-type flies showed less robust proboscis extension to capsaicin, EtOH and quinine compared with sucrose. Inactivation of pain sensation led to loss of aversion to capsaicin. The independent samples *t*-test was used to assess the mean deviation; n = 7. Mean ± S.E.M.; symbols: NS p > 0.05; *p < 0.05; **p < 0.01; ***p < 0.001.

Given that females continue to seek preferred sites for oviposition after one egg laying event, their oviposition preference might be influenced by the positioning and feeding propensity for capsaicin. In tracking the positions of OR flies during oviposition, we found that females were robustly repelled from capsaicin, with a positional index of −0.18 (Fig. 2a). Additionally, the larvae of OR females were repelled from capsaicin on the surfaces of agar plates (Fig. 2b). The consistency of ovipositional and positional repellence suggests that capsaicin acts as a deterrent that naturally repels *Drosophila*. We further sought to explore the feeding preferences for capsaicin by using proboscis extension assays. The results showed that OR females showed aversive behavior to capsaicin (Fig. 2c). Concomitantly, feeding aversion was observed for quinine and ethanol treatment compared with H_2_O mock treatment (Fig. 2c). Together, these results demonstrated that capsaicin is a stimulant that simultaneously triggers ovipositional, positional, and feeding repellence.

### The nociceptive system mediates ovipositional avoidance in response to capsaicin

**Figure 3:**
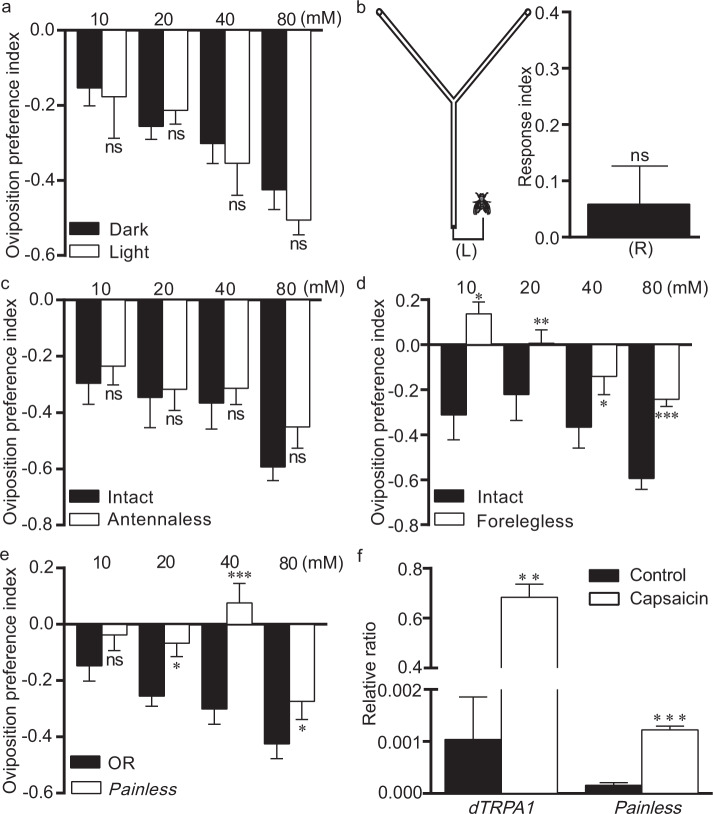
The pain-sensing system mediates ovipositional aversion to capsaicin. (**a**) Vision is dispensable in the ovipositional repellence of capsaicin. For vision, wild-type flies in darkness and light were used. n = 6–15. (**b**) Olfaction is not required for ovipositional aversion to capsaicin. Schematic drawing of the Y maze olfactory assay used for olfaction assays with capsaicin (L) and the response index for capsaicin in the Y maze olfactory assay (R). (**c**) Olfaction is not required for ovipositional aversion to capsaicin. Wild-type females with surgically removed antennae were used, and the oviposition index was evaluated. n = 6. (**d**) Gustation/nociception is required for ovipositional aversion to capsaicin. The forelegs of flies were surgically ablated, and the oviposition index was quantified. n = 6. (**e**) Nociception mediated the ovipositional avoidance of capsaicin. (**f**) Capsaicin activated the expression of pain-associated genes in wild-type flies. n = 6–15. The independent samples *t*-test was used to assess the mean deviation within conditions. Mean ± S.E.M.; symbols: NS p > 0.05; *p < 0.05; **p < 0.01; ***p < 0.001.

*Drosophila* ranks egg-laying sites via sensory modalities^22^, including vision^23^, gustation^24^, olfaction^14^, and nociception^25^. Next, we attempted to identify potential sensory modalities responsible for the ovipositional aversion to capsaicin. First, we tested the role of vision by allowing flies to lay eggs in darkness. The OR flies were still repelled from oviposition in food halves with capsaicin, and the OI in darkness did not significantly differ from that in light (Fig. 3a). Hence, it is unlikely that vision makes a critical contribution to the ovipositional avoidance to capsaicin. Given that capsaicin has volatile properties, we next examined whether olfaction might be required for this ovipositional avoidance by using the Y-maze assay^14^ (Fig. 3b, L). However, the OR flies displayed no bias to capsaicin, with a response index of 0.05 (Fig. 3b, R), indicating that the olfactory system was not required for the ovipositional repellence from capsaicin. To verify these results, we impaired olfaction by surgically dissecting the primary olfactory organs, the third antennal segments. Indeed, antennaectomized OR females still displayed egg-laying aversion to capsaicin (Fig. 3c). Thus, olfaction is not essential for the ovipositional aversion to capsaicin. Capsaicin elicits a burning pain by activating specific receptors (nociceptor) on sensory nerve endings. Because a subset of sensory neurons are embedded within the fly forelegs, we surgically removed the foreleg tips of OR flies. In those flies, the avoidance to capsaicin was markedly lower than that in intact OR flies (Fig. 3d), indicating that pain-sensing neurons were necessary for this avoidance. The forelegs also contain gustatory bristles that guide flies to select hospitable zones to deposit eggs. To rule out the potential roles of gustation, we next sought to identify the nociceptive receptors that regulate egg-laying aversion to capsaicin. We found that *painless* mutants showed lower avoidance to capsaicin at concentrations below 40 mM than wild-type flies (Fig. 3e). Moreover, the positional and feeding avoidance of *painless* mutants was completely impaired (Fig. 2a–c). Collectively, our results suggest that nociception is required for ovipositional aversion to capsaicin.

To verify that capsaicin triggered the transcription of pain-associated genes, we further quantified the transcriptional level of *painless* and *dTRPA1*^26^ (a nonselective cation channel) in capsaicin-treated guts of OR flies through quantitative PCR. As anticipated, our data showed that levels of both *painless* and *dTRPA1* post capsaicin treatment were higher than those in the control group (Fig. 3f). Therefore, we concluded that capsaicin activates the nociceptive modality that mediates the ovipositional avoidance in response to capsaicin in *Drosophila*.

### Capsaicin hinders the growth of *Drosophila* progeny

**Figure 4:**
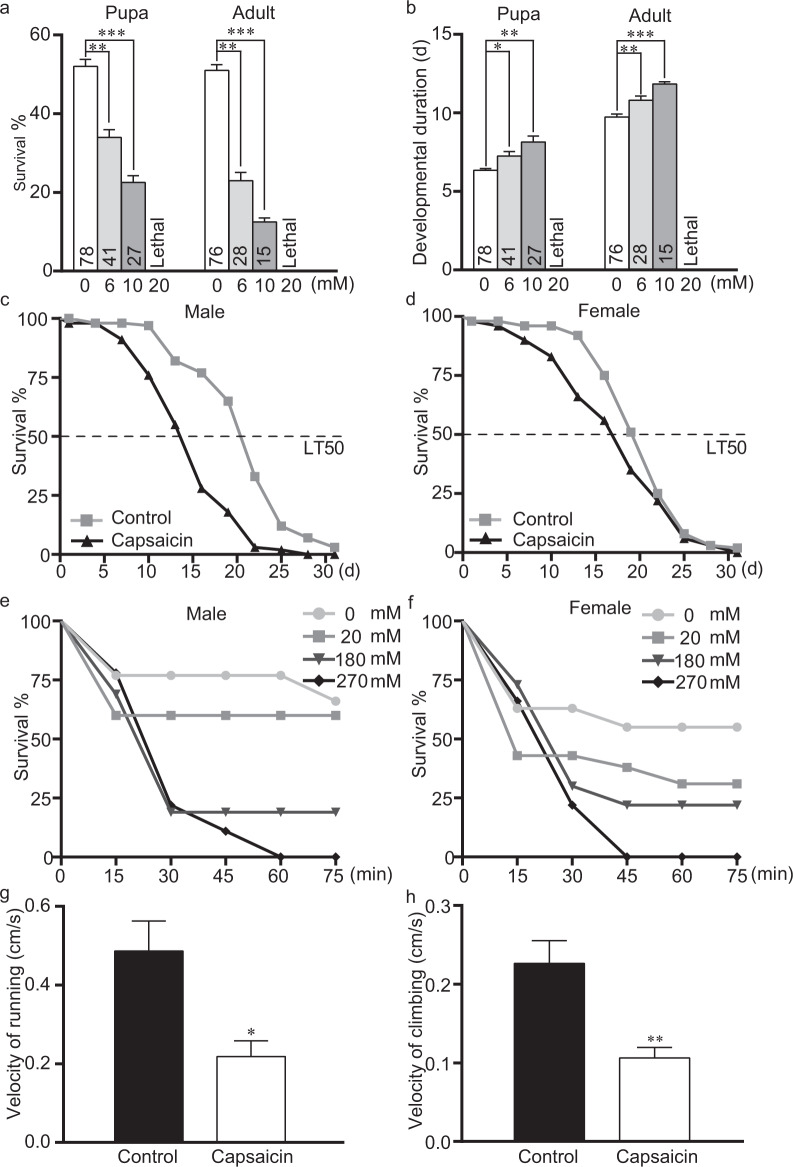
Capsaicin negatively affects *Drosophila* health and lifespan. (**a**) Capsaicin impaired the survival of progenies. Fly eggs were transferred to fly food with different doses of capsaicin, and the percentage of progenies that pupated and eclosed was assessed, respectively. 3 replicates. (**b**) The timing of pupa formation and emergence of adult progenies. The numbers of pupa formation and adult emergence were recorded each day post egg laying. 3 replicates. (**c,d**) The lifespan of flies was shortened by oral capsaicin. 80 mM capsaicin was used. n = 20, 4 replicates. (**e,f**) The lifespans of flies were decreased by contact capsaicin. n = 5, 4 replicates. (**g,h**) Capsaicin decreased the locomotion of flies. Flies were treated with capsaicin, and the velocity of climbing and running was assayed. 80 mM was used. n = 5, 4 replicates. The independent samples *t*-test was used to assess the mean deviation of each column. Mean ± S.E.M.; symbols: NS p > 0.05; *p < 0.05; **p < 0.01; ***p < 0.001.

In nature, *Drosophila* populations deposit eggs in suitable sites to enhance the survival of their offspring^10^. To investigate how capsaicin affects the development of progeny, we treated eggs with different doses of capsaicin. The survival rates of pupae and adults were lower after treatment with capsaicin (Fig. 4a), whereas the animals survived normally in control food. For instance, the survival percentages of pupa and adult formation from eggs declined to 22% and 12% in food with 20 mM capsaicin, whereas the corresponding percentages were 52% and 51% in control food. Furthermore, the pupation and eclosion durations of flies were prolonged with capsaicin treatment compared with the control treatment (Fig. 4b). For example, the duration of formation of pupa and adults from eggs was 6.3 and 9.7 days in control food, respectively. Nevertheless, the corresponding durations were prolonged to 8.1 and 11.8 days in food containing 10 mM capsaicin. Overall, our data support that capsaicin diminishes the fitness of progeny, thus reflecting a strategy of ovipositional avoidance in wild flies.

### Capsaicin decreases the lifespan and climbing behavior of adult flies

Capsaicin widely repels insects^8^ and therefore may directly exert adverse effects on adult OR flies. Indeed, the survival of these flies was dramatically less than that of control flies (Fig. 4c,d), indicating that capsaicin was toxic to flies. Notably, male flies were more susceptible to capsaicin than females, partially explained by the fact that bodyweight of males is lighter than that of females. Capsaicin also serves as a contact insecticide that has adverse effects on insect survival. Indeed, abdomen-contacted capsaicin caused greater lethality in females (Fig. 4e,f). Coordinated locomotion is required for fundamental activities of life. As expected, the results showed that capsaicin impaired running and climbing behaviors (Fig. 4g,h). Collectively, our results demonstrated that capsaicin decreased the fitness of both *Drosophila* parents and progenies.

### Capsaicin disrupts intestinal integrity in *Drosophila*

**Figure 5:**
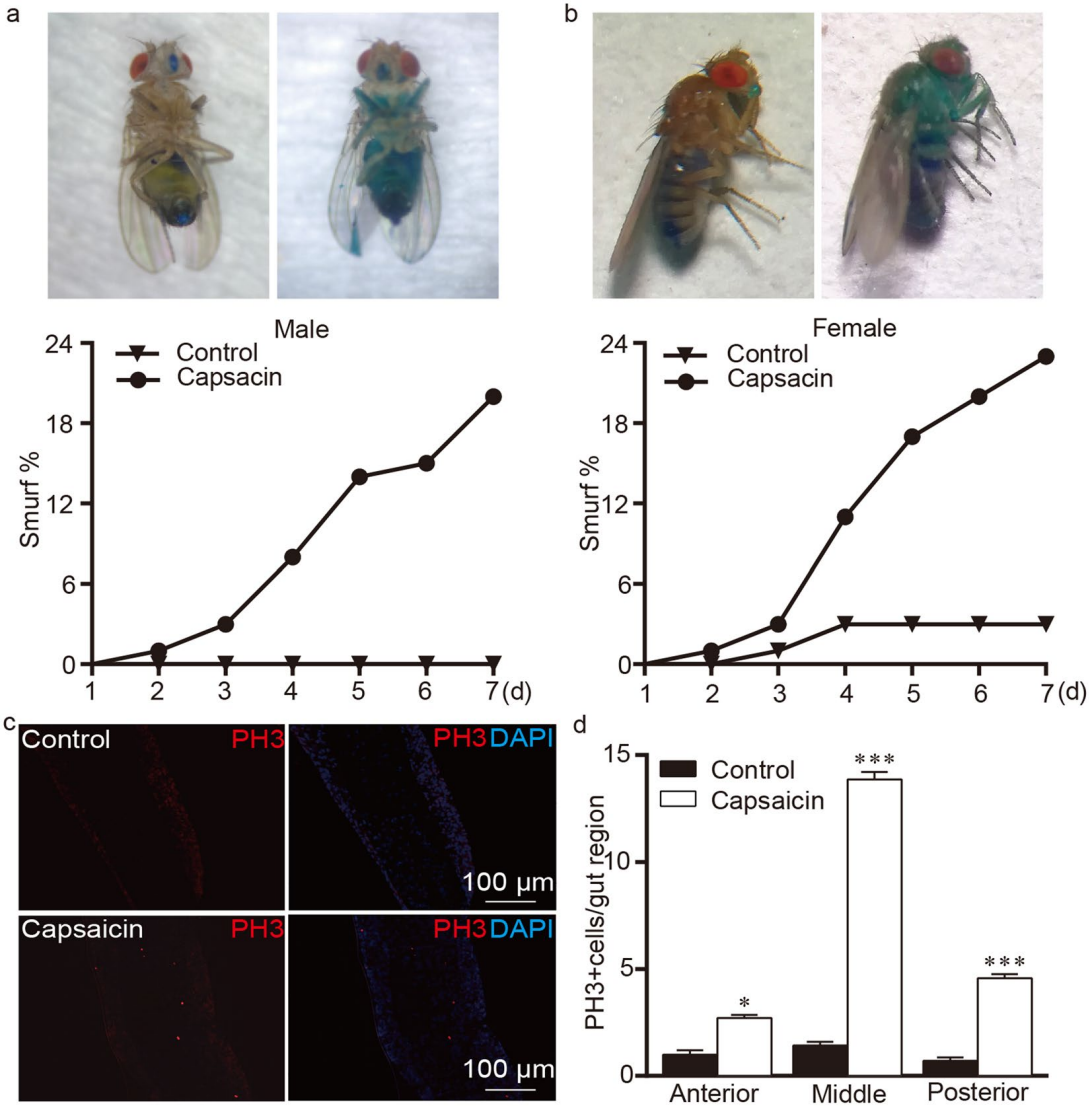
Capsaicin disrupts the integrity of intestines. (**a,b**) The representative images of “smurf” flies (top) and the percentage of “smurf” in flies (below). “Smurf” flies displayed intestinal barrier dysfunction by blue dye permeation throughout the body. 80 mM capsaicin was used. n = 12. (**c**) Capsaicin stimulated the proliferation of intestinal stem cells in the midgut. Representative images in which mitosis is indicated by red staining with PH3, and nuclei are stained blue with DAPI. (**d**) Quantification of PH-3 positive cells in the gut regions of control and capsaicin exposed flies. The anterior gut, midgut, and posterior gut are parts of the gut regions. 80 mM capsaicin was used. n = 8, three replicates. The independent samples *t*-test was used to assess the mean deviation of each column. Values represent mean ± S.E.M. **P < 0.01; ***P < 0.001.

Wild-type flies may ingest capsaicin-containing food that adversely affects their digestive tracts. Using an unabsorbable blue dye, we assayed for loss of intestinal barrier function, as previously described^18,27^. We found that the percentage of loss of the intestinal barrier was higher in capsaicin-treated flies than in control flies (Fig. 5a,b), suggesting that capsaicin caused dye leakage into the hemolymph and consequently all tissue. Intestinal stem cells generate progeny to replenish cell loss induced by acute injury; therefore, we further assessed the number of dividing cells with an anti-phosphorylated histone 3 (PH3) antibody to determine mitotic activity^28^. The number of PH3-positive cells was significantly greater in flies treated with capsaicin than in control flies (Fig. 5c,d). Overall, our data suggested that capsaicin disrupts intestinal integrity of OR flies.

### Capsaicin triggers oxidative innate immunity defense

**Figure 6:**
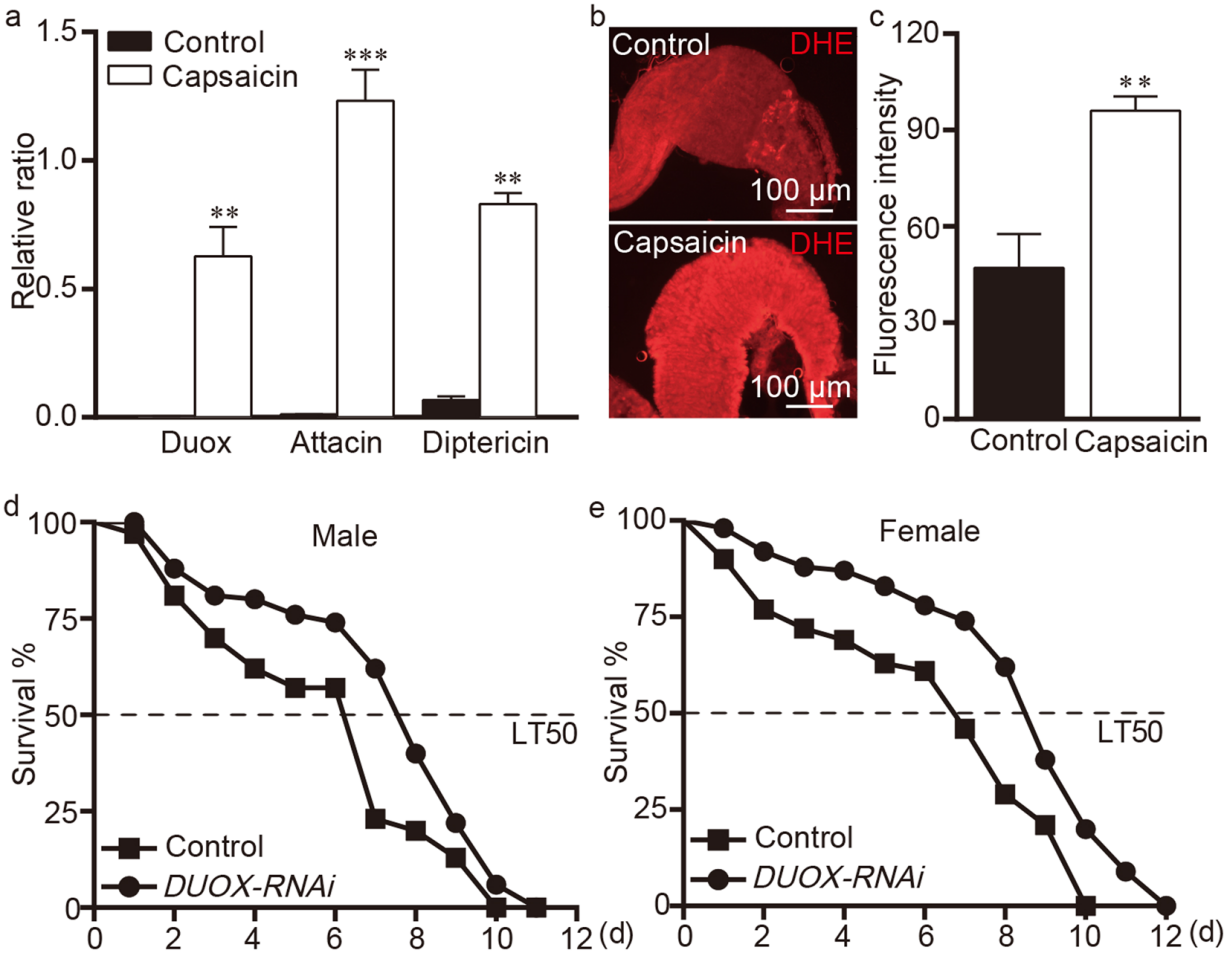
Capsaicin triggers oxidative innate immunity. (**a**) Capsaicin activated the expression of target genes associated with oxidative innate immunity. *DUOX*, *Attacin*, and *Diptericin* mRNA levels for capsaicin-associated wild-type flies. 80 mM capsaicin was used. (**b,c**) Regulation of ROS activity in the gut. ROS activity was measured with DHE. 80 mM capsaicin was used. n = 6, three replicates. (**d,e**) Survival of *Duox*-silenced adult flies challenged with capsaicin. 80 mM was used. n = 150; The independent samples *t*-test was used to assess the mean deviation of each column. Values represent mean ± S.E.M. **P < 0.01; ***P < 0.001.

The expression of the antimicrobial peptides *attacin* and *diptericin* was elevated in the guts of OR flies after capsaicin treatment (Fig. 6a). Dual oxidase (*Duox*) is a member of the ROS-producing nicotinamide adenine dinucleotide phosphate (NADPH) oxidases^29^. As expected, the expression of *Duox* was significantly higher in the midgut in capsaicin-treated flies than control flies (Fig. 6a). Moreover, the ROS levels were higher in capsaicin-treated OR flies than control OR flies (Fig. 6b,c). To validate the role of ROS in mediating the response to capsaicin, we knocked down *Duox* in flies with the GAL4/UAS system^20^. The *Duox* silencing markedly decreased capsaicin’s toxicity to flies (Fig. 6d,e). Taken together, these results suggest that capsaicin triggers oxidative innate immunity in the midgut.

## Discussion

Egg laying selection is used to detect aversion toward compounds that are toxic to both larvae and adults. In this work, we showed that *Drosophila* females exhibit a vigorous ovipositional aversion to capsaicin through the nociceptive neurons expressing the *painless* gene. In addition, capsaicin decreases the fitness of both offspring and parents through intestinal dysplasia and hyperactivation of oxidative innate immunity. Overall, our findings regarding capsaicin avoidance suggest an adaptation promoting the survival and fitness of both parents and offspring^30,31^, providing insight into the resource requirements and ecological behaviors of *Drosophila* species.

Because of their limited mobility, larvae are vulnerable to predators and toxicants. Female flies must select preferred oviposition sites to increase the survival of their offspring^11^. As such, selecting an appropriate site for oviposition has become an innate behavior in *Drosophila* females. For herbivorous insects, *Drosophila* feed on fruits and engage in a persistent battle with host plants^32^. However, many plants use a chemical defense system to generate many secondary metabolites, including capsaicin^4^. For example, capsaicin has been shown to hinder feeding by many invertebrates^32^. Recent studies, including our work, suggest a general theme in which *Drosophila* is robustly repelled from many toxicants, including harmful molds^12^. Ovipositional deterrents have not previously been tested for the onion fly with host models^6^. Flies at all times must balance the benefits and threats of progeny fitness and their survival, because survival and reproduction strategies are used in the context of systemic ecology^10^. Interestingly, a previous study has found a feeding preference for capsaicin when wild-type flies are given a choice between capsaicin-laced sucrose and sucrose alone^4^. The difference of these results may be explained by the differences in capsaicin concentrations. Because capsaicin affects thermoregulation of insects through TRPA receptors, we speculate that low concentration of capsaicin could provoke excitement, but high concentration would activate the defense reaction. Indeed, capsaicin in high concentrations has known insecticidal properties^6,8^, potentially resulting in fly feeding avoidance of capsaicin. Further exploration of the subtle interactions involved will be of great interest. Of note, it is unlikely that the ovipositional avoidance of capsaicin stems simply from the feeding and positioning avoidance of capsaicin (Fig. 2). The oviposition preference does not, in fact, constantly correspond to a positional preference, because *Drosophila* balances multiple environment cues, even competing behavioral drives before behavioral output^22^. For example, acetic acid is an ovipositional stimulant that flies otherwise avoid^21^. Our results showed that up to 200 mM capsaicin efficiently decreased the survival of flies in a contact toxicity bioassay (Fig. 4e,f). Therefore, it is postulated that female adults can endure capsaicin in sites with concentrations of capsaicin (20–80 mM) when they are laying eggs. In addition, female flies laid comparable numbers of eggs in the absence or presence of 40 mM capsaicin (Fig. 1e), suggesting that low concentrations of capsaicin do little harm to adult females. Overall, our findings suggest that female flies make an independent decision to avoid ovipositing in response to capsaicin.

Nociception is caused by physical (heat, cold, and pressure) or chemical (acid, irritants, and inflammatory mediators) stimuli^33^. Nociceptive sensory neurons detect these stimuli and activate neural circuits that elicit stereotyped escape responses^34^. Our findings showed that the ovipositional aversion toward capsaicin is mediated by the activation of the nociceptive neurons expressing the *painless* gene (Fig. 3). In *Drosophila*, the *painless* gene—an evolutionary homolog of the mammalian isothiocyanate receptor *TRPA1/ANKTM1*^4^—is required for the foraging avoidance of isothiocyanates, because isothiocyanates from wasabi and capsaicin can give rise to a burning sensation^25^. Painful or threatening experiences trigger escape responses that are guided by nociceptive neuronal circuitry^3^. Efficient and rapid escape behavior in reaction to threatening sensory stimuli is vital for defense and ultimately survival. *Drosophila* larvae must forage for food while avoiding noxious stimuli and predators, such as parasitoid wasps^14^. Capsaicin is derived from the genus *Capsicum*, its receptor, transient receptor potential vanilloid subfamily member 1 (TRPV1), is localized to many human organs^34^, including the brain, intestine, liver, pancreas, lung, and kidney. Nociception is the encoding of a noxious stimulus and its transduction into electric signals. Noxious stimuli are detected by nerve endings found throughout the body and originating from the PSNs. The activation of TRPV1 induces cation (Ca^2+^) influx, which further triggers voltage-gated sodium channels and generates an action potential^26^.

Animals across multiple species must navigate a complicated and ever-changing environment for survival and propagation. For instance, oxidative stress induces immune activation in both *Drosophila* and humans^35^. Capsaicin ingested from the environment transiently interacts with intestine epithelia by passing through the alimentary flowing stream. Consequently, capsaicin results in intestinal epithelium impairment and intestinal barrier dysfunction in adult flies (Fig. 5). Intestinal barrier dysfunction is a good predictor of stress-induced mortality. To adapt to acute stress in the gut, animals have evolved to modulate their innate immunity to achieve intestinal homeostasis^35^. Although immunity is critical for host fitness in response to stress, the specific signaling pathways through which capsaicin triggers immunity are not yet entirely clear. This study was based on the well-characterized *DUOX*-dependent intestine immunity in flies. We found that capsaicin resulted in *DUOX*-dependent intestinal immune activation (Fig. 6). *DUOX*, a member of the NADPH oxidase family, functions as the first line of defense against intestinal stress by producing ROS. *DUOX*-dependent ROS are considered to be involved directly or indirectly in epithelial turnover through activation of intestinal stem cells in the presence of stress^36^. In lines with previous findings^37^, capsaicin triggers the immune pathway via de novo production of AMPs. Thus, capsaicin-induced *DUOX*-activating signaling exerts a pronounced effect on the lifespan and health of *Drosophila*.

Using *Drosophila*, we investigated an ecological phenomenon whereby capsaicin repels *Drosophila* females from oviposition. Consistent with this finding, capsaicin decreases the fitness of *Drosophila* through intestinal dysplasia. Future research will facilitate more comprehensive understanding of the relationship between the molecular pathology and ecological behaviors of *Drosophila*.

## Materials and Methods

### Fly husbandry and stocks

All fly stocks were raised at 25 °C, 60% humidity in a 12/12 h light/dark cycle on standard cornmeal-yeast-sucrose food unless otherwise noted^10^. Oregon R and Canton S strains were used as wild-type flies. *NP3084* and *DUOX* mutants were a gift from Prof. Gao Guanjun (Shanghai Technology University)^20^. *Painless* mutants and *D. yakuba* were from the Core Facility of *Drosophila* Resource and Technology, Shanghai Institute of Biochemistry and Cell Biology, CAS, China.

### Oviposition preference assays

The two-choice apparatus was assembled with a transparent 80-mm column with a 60-mm Petri dish at the bottom^10,21^. The two-choice dishes were generated by evenly dividing food into halves with a razor blade. In brief, capsaicin (MACKLIN) was dissolved in absolute alcohol, and diluted capsaicin-containing solutions (10 mM, 20 mM, 40 mM, and 80 mM) were added to the surfaces of the egg-laying substrate. The two-choice dishes were placed in air for 3 h to volatilize the alcohol. For each test, 25 newly eclosing females were collected and mated for 3 days in the presence of yeast paste. Flies were gently transferred into assay cages without CO_2_ anesthesia and were allowed to lay eggs for 8 h in darkness. To assess oviposition preference, we counted the number of eggs on each half and determined the OI as follows: [OI = (number of eggs laid on experimental food – number of eggs laid on control food)/total number of eggs laid]. For one-forced assays, 25 mated females were transferred to cages totally with capsaicin-containing substrate, where they were allowed to lay eggs for 12 h. Flies were removed, and laid eggs were counted. Egg laying was calculated by dividing the number of eggs by the total number of live females.

### Position preference assays

The two-choice apparatus was the same as that used in oviposition preference assays^10,21^. For positional preference assays, 200 flies were transferred to cages. The number of flies on each food half was counted at 10-min intervals for 2 h via a camera. The number of flies was summed and averaged, and a position index (PI) was calculated as follows: [PI = (number of flies on experimental food – number of flies on control food)/total number of flies on food].

For surgeries, females were anesthetized with CO_2_ on a pad, and the antennae and tarsi were removed with fine forceps. Flies were allowed to recover for 48 h before testing.

### Feeding preference assays

To assay feeding preferences, each compound was dissolved in 5% sucrose solution as previously described^14^. Briefly, files were collected on the day of eclosion and kept in standard corn meal food for 3–7 days at 25 °C. Before the feeding assays, females were starved for 24 h at 25 °C in vials with a water-saturated Whatman filter paper. Flies were anaesthetized by chilling on ice, mounted by their backs/wings on a microscope slide using double-sided Scotch tape and allowed to recover for 1 h at room temperature. Taste solutions were delivered with a 10 μl pipette to legs for up to 20 s, and the time of proboscis extension was examined. The feeding index (FI) was calculated as follows: FI = the total time of proboscis extension/20 s ×100%.

### Contact toxicity bioassay

The contact toxicity bioassay was performed as previously described^38^. Briefly, files were collected on the day of eclosion and kept in standard corn meal food for 5 days at 25 °C. Flies were anaesthetized with CO_2_, mounted by their wings on a microscope slide using double-sided Scotch tape and allowed to recover for 30 min at room temperature. Each compound was dissolved in 75% EtOH solution, and 10 μL solutions with different concentrations of capsaicin were evenly spread on their abdomens (n = 5, 4 replicates). Flies without leg movements were designed as dead flies every 15 minutes, and the survival rate was calculated.

### Survival ratio assays

In feeding experiments^39^, flies were starved at 25 °C for 4 h before transfer to cages containing a filter paper with 1 ml capsaicin–sucrose. Capsaicin feeding was performed at 25 °C. For viability analysis, we used 20 flies per vial, and the experiments were repeated four times. Flies were transferred to new cages with new capsaicin–sucrose filter paper discs every 24 h and counted every day. The percentage of survival was calculated.

### Dye penetration test (smurf)

The intestinal barrier dysfunction assay was conducted on flies with starved at 25 °C for 5 h. The feeding experiment was performed as described above. Twenty flies were used in every vial and kept at 25 °C. Flies were first exposed to capsaicin–sucrose filter paper for 5 d and then transferred to new cages with filter paper mixed with 2.5% Erioglaucine (FD&C Blue #1)^18^ and 5% sucrose. For the dye penetration test, 20 wild-type flies were fed on filter paper soaked with capsaicin-sucrose solution and Erioglaucine. A fly was counted as a Smurf when dye coloration was observed outside the digestive tract. Smurf % = number of smurf flies/(number of non-Smurf flies + number of smurf flies) × 100%.

### Development timing assays

The embryos were inoculated into fly food with 0 mM, 6 mM, 10 mM, or 20 mM capsaicin (n = 20–35, 5 replicates). The number of embryos was counted with a stereo microscope. The numbers of pupae and emerging adults were counted everyday following embryo incubation. Developmental timing was calculated with the following formula: T = (T1 × N1 + T2 × N2 + … + Tm × Nm)/(N1 + N2 + … + Nm), where T is the developmental timing; Tm is the number of days to form pupae and adults after egg laying; and Nm is the number of pupae and adults on the m^th^ day^12^.

### Climbing and running assays

Polystyrene vials were prepared by placing a Kimwipe moistened with 5% sucrose at the bottom. Capsaicin solution (80 mM) was delivered to the vials everyday. Flies were treated for 5 d and then transferred to a glass graduated cylinder or a serological pipette (5 ml). Locomotion assays were conducted as described with the following modifications^40^. To analyze climbing ability, all flies were synchronized to be tapped down to the bottom of the cylinder. Climbing was recorded using a video camera, and climbing distances at the 6^th^ second were calculated. To analyze running velocity, 5 flies was gently pounded down to the end of a serological pipette in a dark room. This apparatus was horizontal and perpendicular to the light source 15 cm away. Running was recorded using a video camera, and the time to pass the line at the other end of the serological pipette was calculated.

### Real time-PCR analysis

Flies were challenged with 80 mM capsaicin for 48 h, and the guts were dissected and transferred to cold PBS buffer. A total of 40 guts were homogenized with TRIzol reagent (Invitrogen), and RNA was extracted as previously described^12^. RNA was reverse transcribed with an oligo-dT primer. The primer sequences are available upon request. Expression of these genes was assessed with a Bio-Rad CFX instrument. The ΔCt method was used to analyze data with *rp49* as the reference gene. The relative expression value was calculated with the following formula: △Ct = Ct (target gene) - Ct (reference gene), and the relative expression was equal to 2^−△△Ct^.

### Immunofluorescence staining

The immunofluorescence assay was performed as described in previous studies^28^. The flies were fed with 5% sucrose solution with or without 80 mM capsaicin for 5 d. Guts were dissected in PBS and fixed with 4% paraformaldehyde for 30 min at room temperature. Samples were blocked with blocking solution (PBS with 0.3% Triton X-100, 0.2% goat serum and 0.1% fetal calf serum) for 30 min. Guts were incubated in phospho-histone 3 (EMD Millipore, 1:1000 dilution) overnight at 4°C, washed in PBS supplemented with 0.3% Triton X-100, and incubated in PBS with anti-rabbit secondary antibodies (Invitrogen, 1:1000 dilution) and DAPI (Invitrogen, 1:1000 dilution) for 2 h at room temperature. Samples were mounted with Vectshield and observed under a fluorescence microscope (Leica DM4000).

### *In vivo* detection of reactive oxygen species

ROS production in intestinal epithelial cells was measured with the intracellular ROS-sensitive fluorescent dye dihydroethidium^19^. After capsaicin feeding for 48 h, the midguts of flies were dissected in PBS and incubated in a 5 μM concentration of the intracellular ROS-sensitive fluorescent dye dihydroethidium (hydroethidine) (Invitrogen) for 30 min at room temperature in the dark. Then, the midguts were washed three times with PBS, and the tissues were immediately immobilized with 4% paraformaldehyde for 10 min. Next, the tissues were washed three times with PBS. Subsequently, the gut samples were transferred to a glass slide in a drop of PBS for epifluorescence examination and observed under any fluorescence microscope (Leica DM4000). The intensity of immunofluorescence was quantified in ImageJ software.

### Statistical analysis

All analysis was performed in GraphPad Prism statistical software. Specific statistical tests are noted for individual experiments. Significance testing was conducted via a Student’s *t*-test. Figures show mean ± standard error of the mean (S.E.M.). * equals P < 0.05, ** equals P < 0.01 and *** equals P < 0.001^41,42^.
